# Value of a novel Y-Z magnetic totally implantable venous access port in improving the success rate of one-time needle insertion

**DOI:** 10.3389/fsurg.2023.1241780

**Published:** 2023-09-25

**Authors:** Miaomiao Zhang, Xin Lyu, Juanjuan Wang, Aihua Shi, Yunyun Zheng, Yi Lyu, Xiaopeng Yan

**Affiliations:** ^1^ Department of Hepatobiliary Surgery, The First Affiliated Hospital of Xi’an Jiaotong University, Xi’an, China; ^2^ National Local Joint Engineering Research Center for Precision Surgery & Regenerative Medicine, The First Affiliated Hospital of Xi’an Jiaotong University, Xi’an, China; ^3^ Department of Pulmonary and Critical Care Medicine, Second Affiliated Hospital, Xi’an Jiaotong University, Xi’an, China; ^4^ Zonglian College, Xi’an Jiaotong University, Xi’an, China

**Keywords:** magnetic anchor technique, magnetosurgery, total implantable venous access port, Huber needle, magnet

## Abstract

**Background and objectives:**

A totally implantable venous access port (TIVAP) is a commonly used intravenous infusion device for patients receiving chemotherapy or long-term infusion therapy. To improve the success rate of one-time insertion of the Huber needle, we developed a novel Y-Z magnetic TIVAP (Y-Z MTIVAP), which we produced using three-dimensional printing technology.

**Materials and methods:**

The Y-Z MTIVAP includes a magnetic port body and a magnetic positioning device. For testing, we established four venous port implantation models using the two types of TIVAPs and two implantation depth ranges (≤5 mm and >5 mm). Twenty nurses performed Huber needle puncture with the four models, and we recorded the number of attempts required for successful needle insertion, the operation time, and the operator's satisfaction.

**Results:**

The success rate for one-time needle insertion with the Y-Z MTIVAP was significantly higher than that with the traditional TIVAP at either depth range (100% vs. 75% at ≤5 mm, *p *= 0.047; 95% vs. 35% at >5 mm, *p *< 0.001). With increasing implantation depth, the success rate for one-time insertion was significantly reduced with the traditional TIVAP (75% at ≤5 mm vs. 35% vs. >5 mm, *p *= 0.025), but the success rate with the Y-Z MTIVAP was not significantly affected (100% vs. 95%, *p *= 1.000). The operation time with the Y-Z MTIVAP was significantly shorter than that with the traditional TIVAP at either depth range (both *p *< 0.001), and 90% of operators reported that the Y-Z MTIVAP was superior to the traditional TIVAP.

**Conclusions:**

The theoretical design of Y-Z MTIVAP is feasible, and the preliminary *in vitro* simulation experiment shows that it can significantly improve puncture success rate and shortened operation time.

## Introduction

A totally implantable venous access port (TIVAP) is a subcutaneously implantable and long-term indwelling intravenous infusion device, which was introduced in 1982 by Niederhube (1). Implantation within the central vein avoids peripheral vascular sclerosis, embolism and phlebitis caused by highly irritating drugs at high concentrations and can also effectively prevent damage to the blood vessel wall caused by chemotherapy drugs, providing a reliable solution for patients who require long-term infusion therapy and for cancer patients who need chemotherapy (2, 3). The traditional TIVAP has obvious advantages, but there are some limitations in practice. A study of 175 patients found that the success rate for one-time insertion of the Hong Kong needle was only 36% (4). Therefore, methods to improve the success rate of one-time insertion of the Huber needle are needed.

Magnetosurgery (magnetic surgery, MS) describes the use of specially designed magnetic medical instruments or equipment to apply the “non-contact” magnetic field force between magnetic substances in performing specific functions in clinical diagnosis and treatment. MS is used to achieve such functions as complete tissue compression, organ anchoring, lumen navigation, gap expansion, controllable tracing, and directional drive. MS also can be used for digestive tract anastomosis (5–8), cystostomy (9), establishment of an animal model of tracheoesophageal fistula (10), and vascular anastomosis (11–15). The magnetic anchor technique (MAT) is an important magnetic surgical technique (16) that has been used in laparoscopic surgery (17, 18), thoracoscopic surgery (19) and endoscopic submucosal dissection (20, 21) and offers the advantages of reducing trauma and optimizing surgical operations.

In the present study, we introduce a design scheme for a Y-Z magnetic totally implantable venous access port (Y-Z MTIVAP) based on the MAT and demonstrated the feasibility of the device through *in vitro* simulation experiments.

## Materials and methods

### Ethics statement

The research protocol and all experimental procedures were carried out strictly in accordance with the Guidelines for Care and Use of Experimental Animals issued by the Xi'an Jiaotong University Medical Center. This experimental study was approved by the Experimental Ethics Committee of Xi'an Jiaotong University (Permit number: 2022–1452). To follow the “3R” principle for animal experiments and reduce the use of experimental animals, we used the abdominal wall of experimental pigs sacrificed after use in other studies in our laboratory to perform simulated puncture operations using the Y-Z MTIVAP and the traditional TIVAP.

### Y-Z MTIVAP

**Figure 1:**
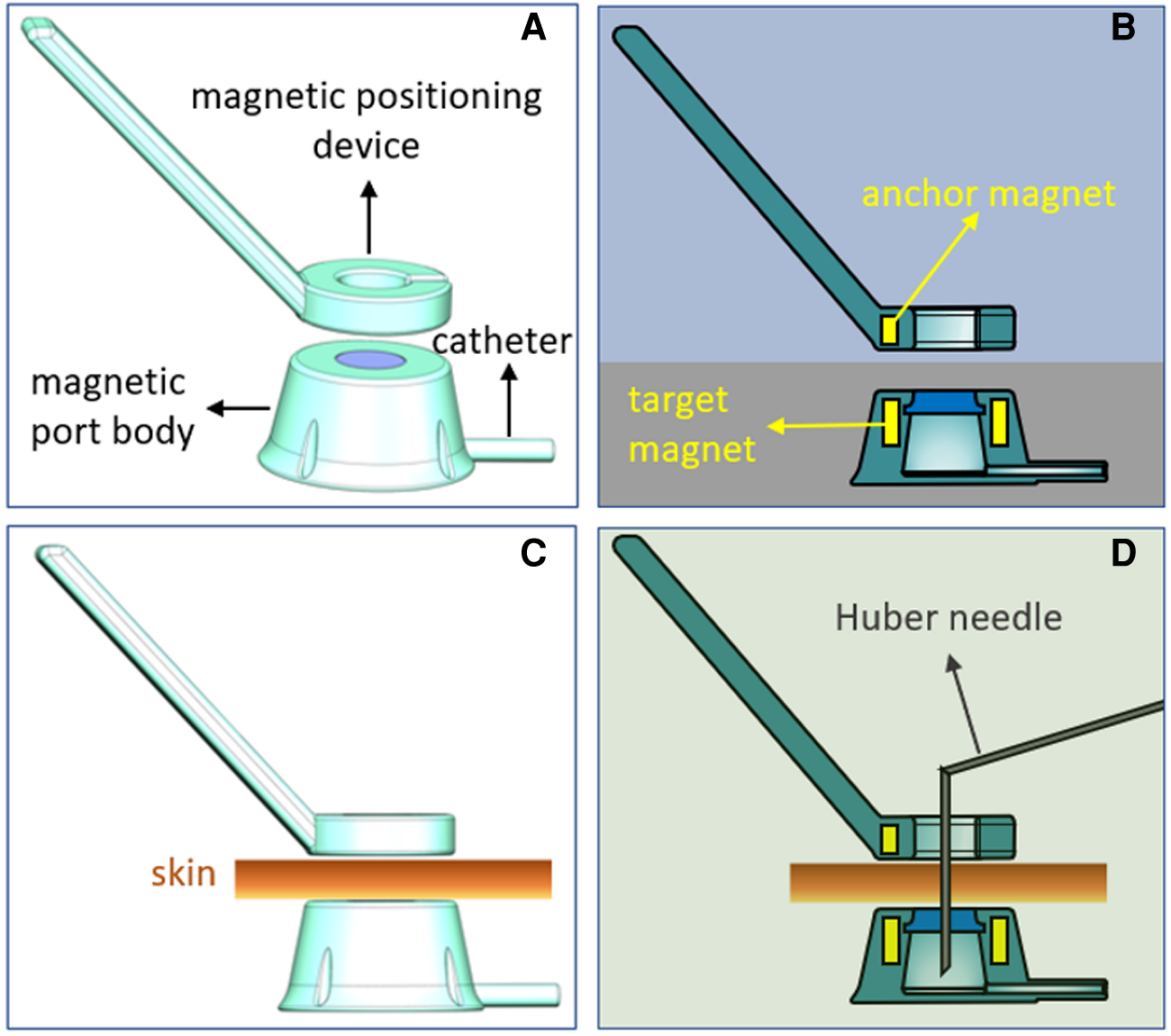
Schematic diagram of the Y-Z magnetic total implantable venous access port (MTIVAP). (**A**) Schematic diagram of the overall appearance of the Y-Z MTIVAP. (**B**) Sectional view of the Y-Z MTIVAP. (**C,D**) Schematic presentations of the Huber needle puncture procedure.

The Y-Z MTIVAP consists of a magnetic port body and a magnetic positioning device. The magnetic port body includes structures such as a silicone diaphragm, port body, connecting conduit and fixing hole. The difference between the Y-Z MTIVAP and the traditional TIVAP is that a magnetic ring referred to as a target magnet is embedded outside the silicone diaphragm of the port body. Another important structure is the spoon-shaped magnetic positioning device. The spoon body is a ring with a gap, and a magnetic ring called an anchor magnet that matches with the target magnet is arranged in the spoon body (Figures 1A,B). Y-Z magnetic port and magnetic positioning device sealed packaging and ethylene oxide sterilization.

#### Application principle

The N pole of the anchor magnet corresponds to the S pole of the target magnet, so that when the magnetic positioning device is close to the magnetic port, they are automatically attracted to each other under the action of magnetic force. For operation, the magnetic positioning device is placed close to the surface skin over the magnetic port body. The target magnet and the anchor magnet are automatically attracted, and the middle hole of the magnetic positioning device and the puncture area (silica gel mold) of the magnetic port body are exactly aligned, providing the operator with a precise puncture area (Figures 1C,D).

**Figure 2:**
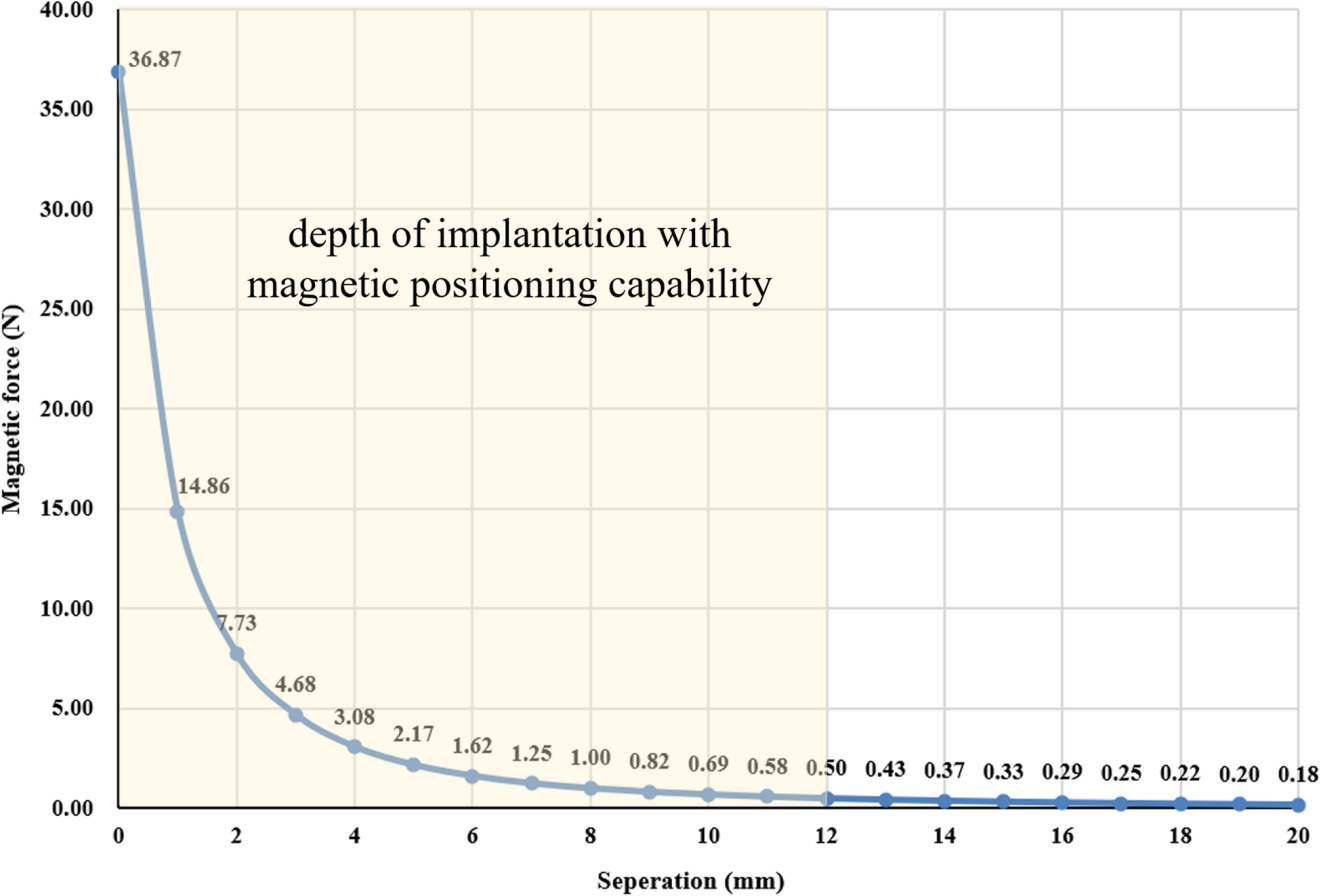
The magnetic force-displacement curve of the anchor magnet and target magnet.

In this study, the target magnet was a ring (outer diameter: 16.8 mm; inside diameter: 12 mm; height: 6 mm), and the magnetic field intensity was 4,350 GS. The anchor magnet was a ring with a 3-mm gap (outer diameter: 16.8 mm; inside diameter: 12 mm; height: 3 mm) with a magnetic field intensity of 3,760 GS. The target and anchor magnet were made of N45 neodymium-iron-boron (NdFeB). The magnetic forces between the anchor magnet and target magnet at distances of 0 mm, 5 mm and 10 mm were 36.87 N, 2.17 N and 0.69 N, respectively. The magnetic force-displacement curve of the anchor magnet and target magnet is shown in Figure 2.

### Study design

**Figure 3:**
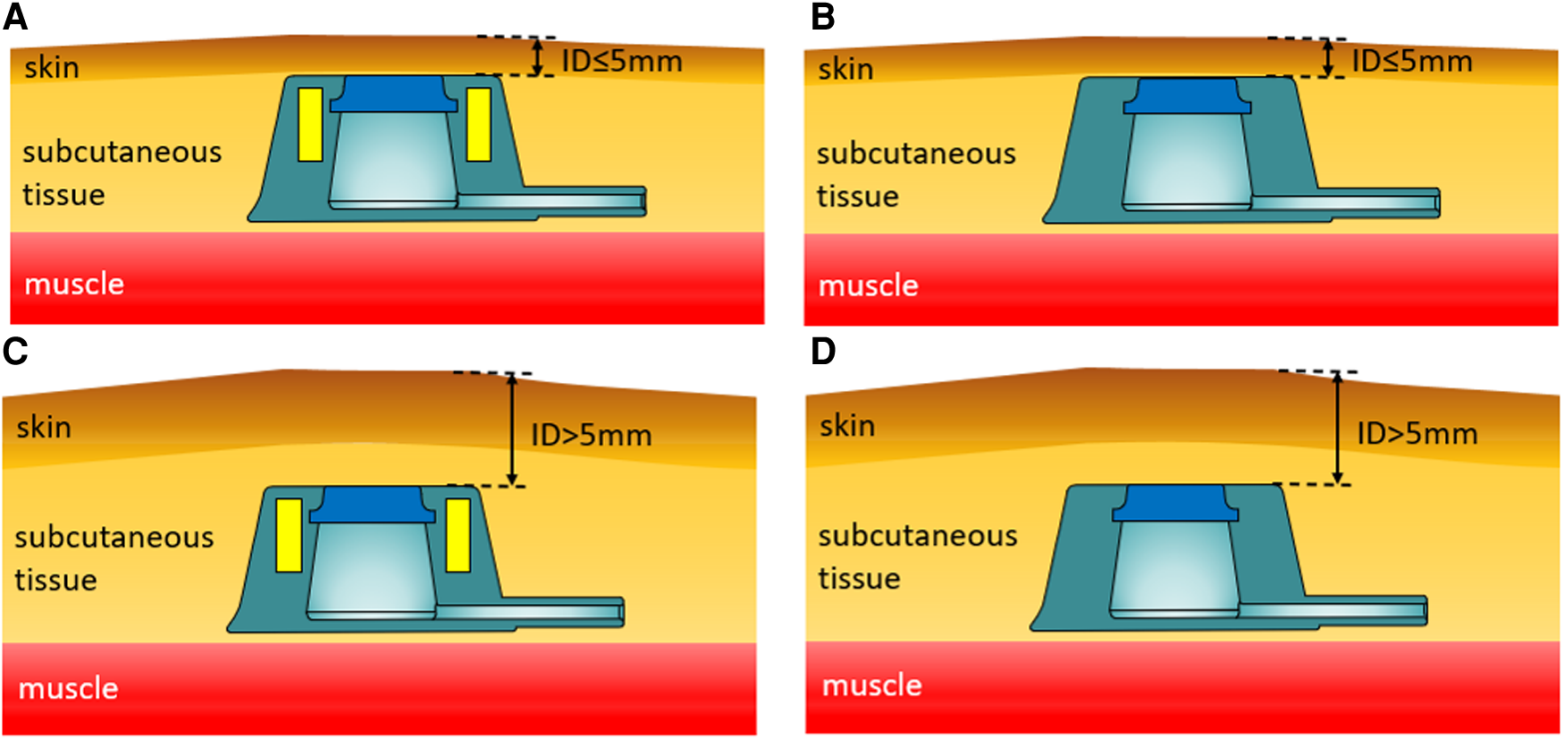
Schematic diagram of four venous access port implantation models. (**A**) Use of the Y-Z MTIVAP with an implantation depth (ID) ≤5 mm. (**B**) Use of the traditional TIVAP with an ID ≤5 mm. (**C**) Use of the Y-Z MTIVAP with an ID >5 mm. (**D**) Use of the traditional TIVAP with an ID >5 mm.

Abdominal wall sections were obtained from experimental pigs and used to prepare an *in vitro* model for venous port implantation. According to the type of implanted venous port and the implantation depth (ID), four *in vitro* models for venous port implantation were established: group A (Y-Z MTIVAP and ID ≤5 mm), group B (traditional TIVAP and ID ≤5 mm), group C (Y-Z MTIVAP and ID >5 mm), and group D (traditional TIVAP and ID >5 mm; Figures 3A–D).

To estimate the study power, the use rate of the success rate for one-time insertion of the Huber needle as the primary endpoint. According to our preliminary experimental data, the success rate for one-time insertion in Y-Z magnetic totally implantable venous access port and traditional totally implantable venous access port were 75% and 35%, respectively. Assuming the first type error as α = 0.05 and deeming a power of 80%, a total operation number is 28 cases (14 cases per group) was calculated adequately.

Twenty nurses performed Huber needle puncture for each of the four venous port implantation models sequentially. We recorded the number of attempts required for successful Huber needle insertion as well as the operation time and the operator's satisfaction. After the Huber needle was inserted into the TIVAP, diluted methylene blue solution was injected. The insertion was deemed successful if the blue liquid flowed out of the subcutaneous catheter smoothly. Operating time was recorded from the start of the operation to the successful insertion of the Huber needle. Each nurse rated their satisfaction with the operation as good, fair or bad according to his/her own experience.

### Statistical analysis

SPSS statistical 20.0 software was used for data analysis. Continuous variables that followed a normal distribution are reported as the mean ± standard deviation (SD) and were compared using analysis of variance tests. The comparison of the success rate for one-time insertion of the Huber needle between Y-Z port and traditional Port was performed by Chi-square test. Variables that followed an abnormal distribution are reported as median [interquartile range (Q1, Q3)] and were compared using nonparametric tests. All hypothesis tests were two-sided, and *p* values < 0.05 were considered statistically significant.

## Results

**Figure 4:**
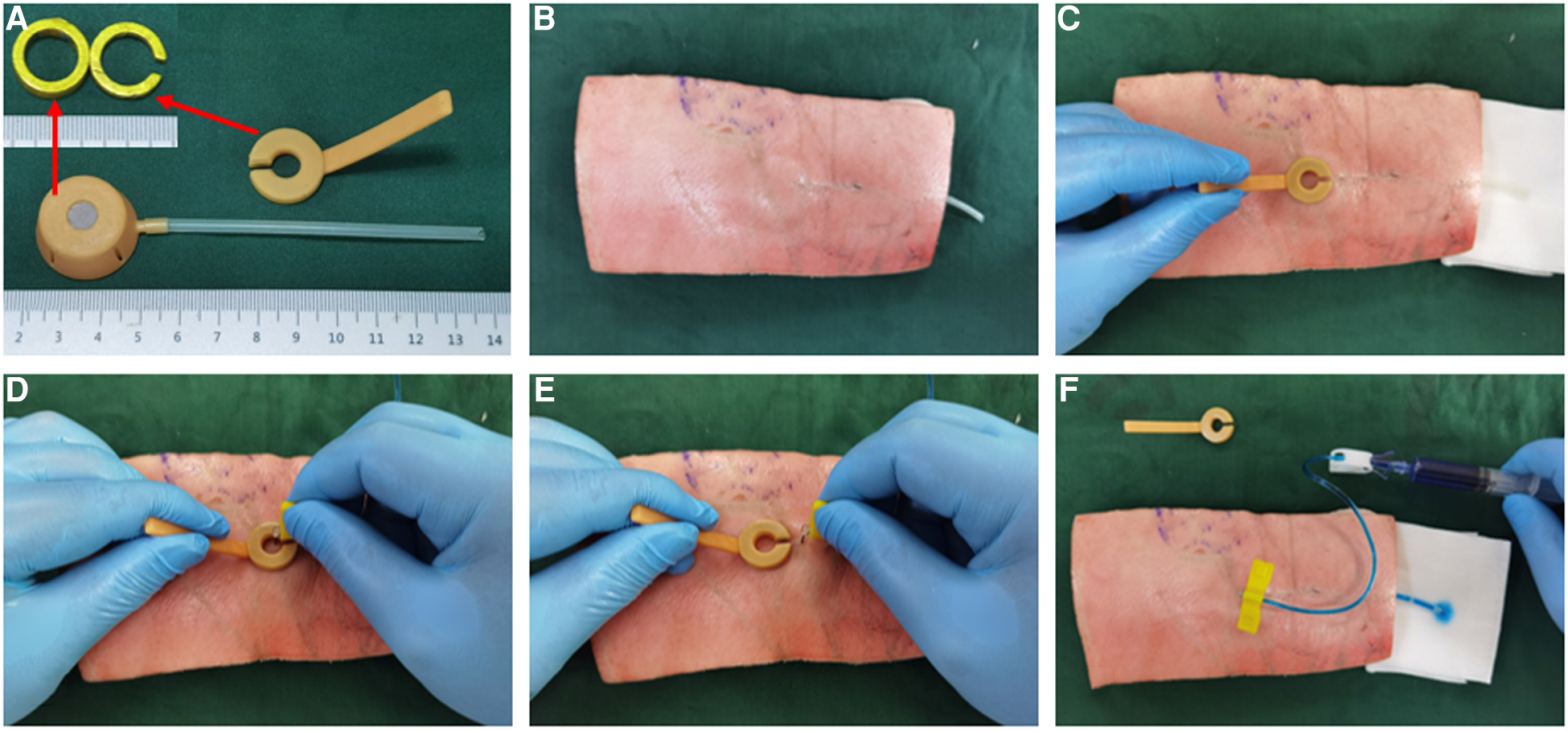
In vitro simulated operation of Y-Z MTIVAP implantation. (**A**) Image of the three-dimensionally printed Y-Z MTIVAP. (**B**) Y-Z MTIVAP implantation in the isolated abdominal wall of an experimental pig. (**C**) Magnetic positioning device attracting the magnetic port to determine the puncture area. (**D**) Insertion of the Huber needle in the circular area of the magnetic positioning device. (**E**) Removal of the magnetic positioning device. (**F**) Verification of successful puncture with the Huber needle.

**Figure 5:**
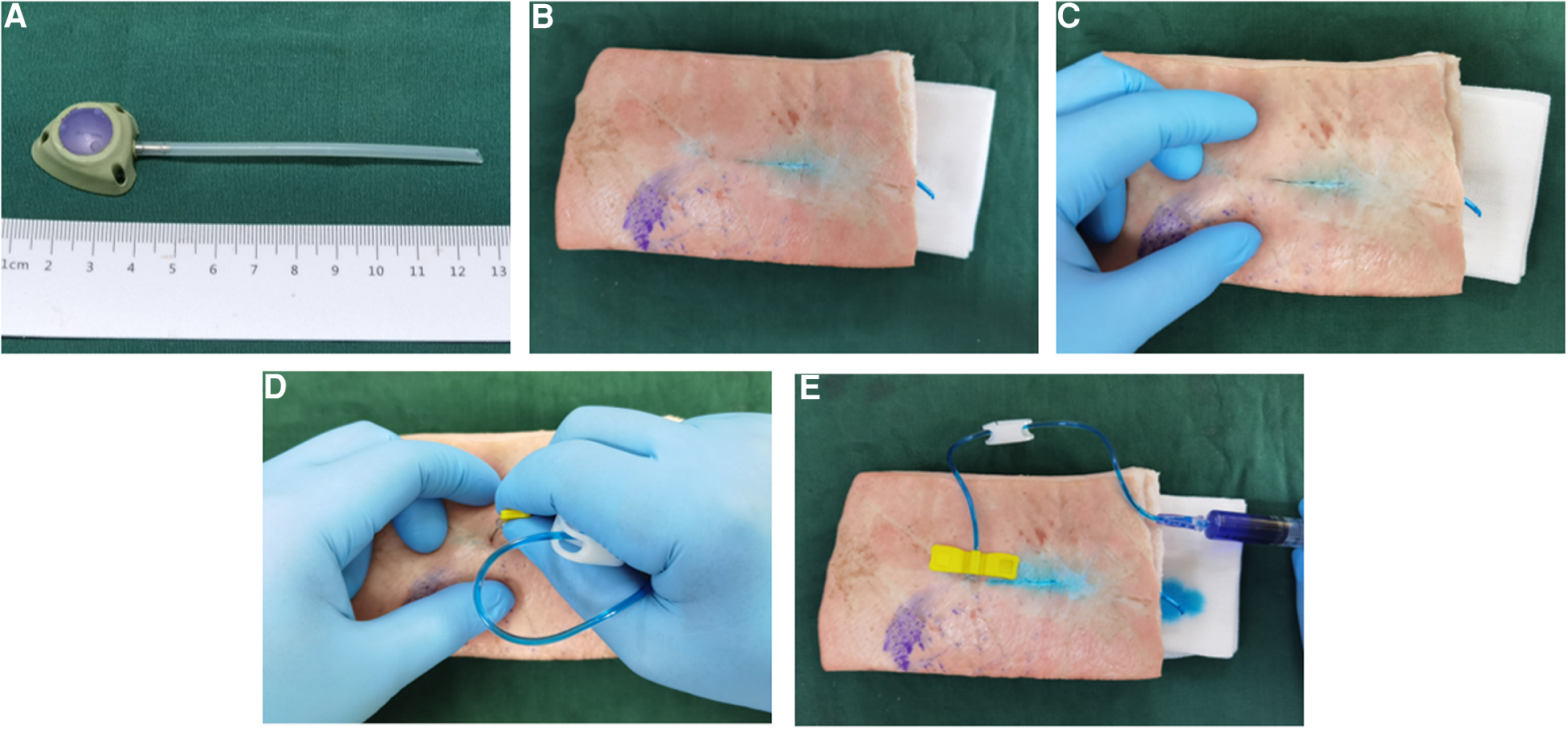
In vitro simulated operation of traditional TIVAP implantation. (**A**) Image of the traditional TIVAP. (**B**) Traditional TIVAP implantation in the isolated abdominal wall of an experimental pig. (**C**) Three-point positioning method to determine the puncture area. (**D**) Insertion of the Huber needle. (**E**), Verification of successful puncture with the Huber needle.

We successfully produced the Y-Z MTIVAP using three-dimensional printing technology (Figure 4A). Upon implantation of the Y-Z MTIVAP subcutaneously into the isolated abdominal wall of experimental pigs (groups A and C), the magnetic positioning device was close to the skin surface near the magnetic venous port, and the magnetic positioning device was immediately attracted by the target magnet to the skin over the surface of the magnetic venous port. After insertion of the Huber needle into the circular area at the tip of the magnetic positioning device, the magnetic positioning device was removed and methylene blue solution was injected to confirm the successful puncture (Figures 4B–F). In the control groups (groups B and D), a traditional TIVAP was positioned, and the skin was punctured according to the conventional method (Figures 5A–E).

The success rates for one-time insertion in each group are presented in Table 1. Group A had the highest one-time puncture success rate of 100%, and group C had the second highest success rate of 95%. These rates were significantly higher than those in the control groups B (75%, *p_A−B _*_= _0.047) and D (35%, *p_C−D _*< 0.001), indicating that the Y-Z MTIVAP could significantly improve the one-time puncture success rate for the Huber needle. As the venous port ID increased, the one-time puncture success rate decreased significantly with the use of the traditional TIVAP (*p_B−D _*= 0.025), indicating that the ID was an important factor affecting puncture with the traditional device. However, the success rate in group C was significantly higher than that of group D (*p_C−D _*< 0.001), indicating that the Y-Z TIVAP can effectively overcome the difficulty of puncture caused by increased ID.

**Table 1:** Puncture success rates for the different implantation models.

	Group A	Group B	Group C	Group D	*p* value
Number of attempts until successful puncture (*n*, %)					
1	20 (100%)	15 (75%)	19 (95%)	7 (35%)	*p_A−B _*= 0.047
>1	0 (0%)	5 (25%)	1 (5%)	13 (65%)	*p_B−D _*= 0.025
					*p_A−C _*= 1.000
					*p_C−D _*< 0.001
Operation time (s)	22.5 (19.75, 26)	32 (27.75, 39)	22.5 (20, 28)	45 (39.75,58.50)	*p_A−B _*< 0.001
*M (Q1, Q3)*					
					*p_C−D _*< 0.001
					*p_B−D _*< 0.001
					*p_A−C _*= 0.903

The operation times in groups A (22.5 s) and C (22.5 s) were significantly shorter than those in groups B (32 s, *p_A−B _*< 0.001) and D (45 s, *p_C−D _*< 0.001), respectively, indicating that the magnetic positioning device facilitated quick puncture positioning, which can significantly shorten the operation time compared with the traditional three-finger fixation method. With use of the traditional TIVAP, the operation time also increased with a greater ID (*p_B−D _*< 0.001), but the increase in the ID had little effect on the operation time for Y-Z MTIVAP implantation (*p_A−C _*= 0.903). Eighteen of the twenty nurse rated the Y-Z MTIVAP as superior to the traditional TIVAP, with high recognition and acceptance.

## Discussion

The results of this study showed that Y-Z MTIVAP was significantly different from traditional TIVAP in increasing the success rate for the one-time insertion of the Huber needle (*P* = 0.047). The advantage of Y-Z MTIVAP was more obvious when implantation depth increased (*P* < 0.001). At the same time, the success rate for one-off insertion of the Huber needle could be further reduced (*P* < 0.001). It has important clinical significance to relieve the pain of patients and improve the operation convenience of nursing staff.

Studies have shown that the reasons for failure of Huber needle insertion include weight changes, the type of the device, the length of time the port has remained implanted, and the experience of the clinician performing the procedure (4). Therefore, currently, the first successful attempt at Huber needle insertion is often improved by strengthening the technical training of medical staff in clinical practice, while there is no effectively optimized design for the TIVAP device itself. Clinically, the operator often needs to use the thumb, index finger and middle finger to touch and fix the subcutaneously implanted port body, and then they select the central part of the raised skin as the location for inserting the Huber needle. This method of operation is easy for patients with a thinner body or looser skin. However, when the TIVAP must be implanted too deeply, the port access time is extended. Also, when a patient's skin has poor skin relaxation, it is difficult for the operator to accurately locate the silicone in the center of the TIVAP.

Magnetic force is a special “non-contact” field force that is not blocked by most objects. Magnetic force also provides a unique three-dimensional positioning ability. According to these characteristics of magnetic force, we designed the Y-Z MTIVAP. The Y-Z MTIVAP has the following characteristics: (1) The structure design is original and the processing technology is simple. In terms of the design concept, a ring-shaped target magnet is located on the upper portion of the port body and completely surrounding the silicone diaphragm, and the magnetic positioning device used with the venous port has a matching ring-shaped anchor magnet. During the operation, the anchor magnet and the target magnet can be aligned and attracted across the skin. At this time, the area in the positioning ring corresponds to the area where the venous port silicone septum is located, which is the Huber needle insertion area, thereby greatly improving the accuracy of the port needle puncture. (2) The spoon-shaped magnetic positioning device can be easily removed and fixed during the operation. The positioning ring adopts a non-integral structure design, allowing the magnetic positioning device to be smoothly withdrawn from the needle outlet after the Huber needle is inserted. After inserting the Huber needle, the gap in the positioning device allows the magnetic positioning device to be removed. (3) The NdFeB permanent magnet material has excellent magnetic properties. The use of sintered NdFeB as the raw material can maximize the magnetic force with magnets of the smallest size possible.

The Y-Z MTIVAP also has some inherent disadvantages. First, during implantation of the Y-Z MTIVAP, patients must stay away from a strong magnetic field environment, and thus, magnetic resonance imaging (MRI) cannot be performed. Therefore, in clinical practice, implantation of the Y-Z MTIVAP is not suitable for patients who may need to undergo MRI examination in the short term. Second, for patients with implanted electronic devices such as cardiac pacemakers and artificial hearts, the safety of implantation of the Y-Z MTIVAP must be further evaluated. Third, the ID of the port body and the patient's skin laxity remain key factors affecting the insertion of the Huber needle. Compared with the conventional TIVAP, the advantages of the Y-Z MTIVAP are not prominent in patients in whom insertion will be easy, but the advantages of the Y-Z MTIVAP will be prominent in patients in whom insertion will be difficult, which also suggests a suitable patient group exists for use of the Y-Z MTIVAP. Fourth, it can be seen from the magnetic displacement curve that the magnetic force between the anchored magnet and the target magnet gradually decreases with the increase of displacement. When the distance is 12 mm, the magnetic force between them is 0.5 N. At this point, the magnetic positioning device can effectively position the magnetic port body, and the positioning effect decreases with further increase of displacement. Therefore, we suggest that the implantation depth of the magnetic port body should not exceed 12 mm.

In this study, Y-Z MTIVAP was carried out on an *in vitro* pig abdominal wall model, which could not fully simulate the complex and diverse conditions of clinical patients, which is the shortcoming of this study. Of course, Y-Z MTIVAP is still in the design concept and has not officially become a medical product, so at this stage, we are more concerned about its innovative design concept, rather than emphasizing its future clinical value.

In conclusion, we designed the Y-Z MTIVAP according to the principle of the MAT. The device, which has an original design and is easy and inexpensive to produce, can significantly improve the success rate of one-time insertion of a Huber needle, indicating its potentially important clinical application value. Although Y-Z MTIVAP has both advantages and disadvantages, this article adds value to the current knowledge on the innovation design of the totally implantable venous access port. We hope this design idea can inspire and help the peers.

## Data Availability

The raw data supporting the conclusions of this article will be made available by the authors, without undue reservation.
